# Dimensions of the health benefits of wellness tourism: A review

**DOI:** 10.3389/fpsyg.2022.1071578

**Published:** 2023-01-09

**Authors:** Chenmei Liao, Yifan Zuo, Shaogui Xu, Rob Law, Mu Zhang

**Affiliations:** ^1^Shenzhen Tourism College, Jinan University, Shenzhen, China; ^2^School of Physical Education, Shenzhen University, Shenzhen, China; ^3^School of Management, Jinan University, Guangzhou, China; ^4^Asia-Pacific Academy of Economics and Management, University of Macau, Macau, China; ^5^Department of Integrated Resort and Tourism Management, Faculty of Business Administration, University of Macau, Macau, China

**Keywords:** wellness tourism, physical fitness, psychological fitness, quality of life, environmental health, open coding

## Abstract

The benefits of wellness tourism have been recently noted by researchers and industry representatives. This study examined the health dimensions of these benefits posited by a large array of interdisciplinary studies from 2002 up to the present. Open coding was used to conduct an inductive evaluation to classify these health benefits. Results showed four main dimensions, namely, physical fitness, psychological fitness, quality of life (QOL), and environmental health; however, these dimensions need further investigation. Physiological health benefits can also be demonstrated through future experiments, which can further focus on empirical research on the psychological benefits and its overall effect on the QOL. This study contributes to the current literature by providing novel theoretical foundations and subsequently aids practitioners to understand customers better and convey their marketing messages to tourists more effectively.

## 1. Introduction

The concept of “wellness” is holistic; many scholars emphasize the multidimensional nature of personal health, combining physical and mental health, social, and environmental factors. It is explained as a state where one experiences “harmony in mental, physical, spiritual, or biological health in general” through healthy and enriching activities (Kotur, 2022). Wellness has five main conceptualizations: wellness as a state of being or outcome, as a process, as an approach to professional care, as a matter of community, and as a global topic (Stará and Peterson, 2017). With recent rapid economic development bringing in fierce social pressure and competition, involution and an accelerated pace of life has left most people feeling burnt out, leading to an increase in those with subhealth characteristics. Burnout is manifested in three aspects: body, psychology, and interpersonal communication.

The abject disregard of this problem leads to negative effects on work and personal life, reduces the efficiency of social production, and affects social stability. During the past two decades, an increasing turn toward healthy ways and wellness activities occurred (Koncul, 2012). Post-pandemic, various tourists are also anticipated to invest in experiences enhancing their overall sense of wellness. Following the Global Health Institute, the 2019 novel pneumonia epidemic has halted the growth of the global wellness tourism market. However, by the end of 2022, its value will reach 817 billion US dollars.

Tourism enables people to get out of the work environment and experience new things. Participating in leisure activities improves subjective well-being (Fritz and Sonnentag, 2006). Some scholars hypothesize its benefits on the basis of bottom-up tourism benefit spillover theory (Peters, 2000), which suggests that overall life satisfaction is influenced by assessments of various life domains, such as personal health, work, leisure, and family, whereas positive and negative factors in life events influence how individuals assess various life domains.

Some motives or benefits are also unique to specific tourism contexts. Examples include altruism, which is a benefit included in volunteer tourism studies (Brown, 2005). Tourism can be beneficial to a tourist’s health because it provides different physical, psychological, and intellectual benefits. Thus, research on tourists’ health is usually the focus of wellness tourism (Težak Damijanić, 2019). Generally, wellness tourism is a form of tourism that links individual health with social and personal dimensions and considers people’s individual lifestyles.

Recognizing the general trend and exploring the positive effects of health tourism consumption have important theoretical value for improving the industry’s awareness of health tourism strategy, cultivating the positive attitude to life, advocating a kind of healthy lifestyle, and ultimately improving well-being and quality of life (QOL; Pocinho et al., 2022).

Wellness tourism has been growing rapidly, and it has been the subject of intense debate regarding its development, attractive forces, and marketing among others (McCarthy, 2015). As a lifestyle and destination choice for tourists, wellness tourism is not merely guided by the need of relaxation in tourism (Joseph Sirgy, 2019). Wellness tourism experiences have traditionally focused on relaxation, sensory pleasures, enjoyment, and natural environment. However, in recent years, wellness tourism has diversified to offer more immersive and authentic experiences that focused on tourists’ personal growth. The paradigm of wellness tourism’s whole vision has changed from a narrow perspective based on physical health and well-being to a broad vision of holistic health (Dini and Pencarelli, 2022). Recent studies have started to focus more on the benefits of travel experience (Nawijn, 2011). However, most research on this topic is conducted in the fields of organizational behavior and health sciences. Moreover, most studies only discuss the benefits of wellness tourism from a single dimension and do not conduct comprehensive studies on health benefits from a holistic perspective.

Recognizing this gap in the research, this study provides a comprehensive review of the literature pertaining to the impact of wellness tourism experiences to wellness of individuals. Then, on the basis of the results of the literature review, directions for future research are discussed, and recommendations for future wellness tourism destination planning and management are provided.

This study uses open coding and framework analysis to explore the dimensions of the health benefits of wellness tourism. The research interests of the study are justified by the results that an increasing number of tourists spends time and money to enhance their mental and physical well-being. Furthermore, expanding wellness tourist experience studies from a holistic perspective provides a logical connection. In addition, a growing number of tourists is forecasted to invest in experiences to improve their sense of overall wellness. Given the development of wellness tourism, this research not only bridges the gap between the systematic and holistic nature of the existing literature but also becomes instrumental in elevating the quality and variety of wellness tourism offerings.

## 2. The relationship between health and wellness tourism

The definition of “Health” in “*Cihai,*” an unabridged, comprehensive dictionary, includes two aspects. One is that the main organs are free of disease, the body shape is well developed and uniform, and the human body system has good physiological functions and strong physical constitution. Second, it has good resistance to diseases and can adapt to the impact of environmental changes, various physiological stimuli, and pathogenic factors on the body.

Meanwhile, health is defined by the World Health Organization as a state of complete physical, mental, and social well-being, not merely the absence of disease or infirmity. The concept of wellness tourism is well defined. For them, wellness is a state of health featuring the harmony of body, mind, and spirit, with self-responsibility, physical fitness or beauty care, healthy nutrition or diet, relaxation or meditation, mental activity, and environmental sensitivity. It is also regarded as a positive process; in the process of realization, people can perceive and choose a more successful life, promote a positive and optimistic outlook on life, and a comprehensive and balanced outlook on life (Koncul, 2012).

A close relationship exists between tourism and the health of tourists despite tourism’s negative impacts on tourist health given natural disaster and accidents (Heggie, 2009), disease transmission (Chen et al., 2016), and safety risks (Stephens et al., 2005). Meanwhile, some scholars even believe that the sense of well-being brought by leisure forms based on social and recreational purposes is not enough to produce health benefits (Downward and Dawson, 2016).

However, the view that tourism activities have more positive effects on tourists’ health is generally agreed upon. For example, travel experience directly affects the overall life satisfaction of leisure tourists (Neal et al., 1999). Positive effects are also shown on the perceived physical and mental well-being, but these benefits gradually diminish after the trip ends (Chen and Petrick, 2013). Longer tourist stays are therefore likelier to improve the quality of individual leisure life and overall life satisfaction (Neal et al., 2007).

Travel recovery experiences (such as psychological separation from work, relaxation experiences, challenging experiences, and perceived control during vacations) may have positive effects on perceived health (Fritz and Sonnentag, 2006). Some authors used salivary cortisol and chromogranin A (CgA) as endocrinological stress markers to assess the effects on health of participation in a short leisure trip (two nights, 3 days); they found that short-term travel tours probably have beneficial health effects (Toda et al., 2004). Tourism activities help tourists reduce anxiety; the longer the travel time, the lower the anxiety level of tourists (Flaherty and Chua, 2018; Kroesen and De Vos, 2020). Evidently, travel makes people happier (Nawijn, 2011) and is regarded as an effective way to achieve a healthy state (McCarthy, 2015).

Clearly, tourism and health have a natural coupling relationship. General tourism activities are conducive to relaxing the spirit, regulating physical and mental health, and eliminating what modern medicine calls the “third state between health and disease.” Compared with the general form of tourism, the health benefit of forms of tourism with strong health orientation is more evident. Wellness tourism is defined as the sum of all relationships and phenomena from tourism activities undertaken by people with the maintenance and promotion of health as their primary motivation (Mueller and Kaufmann, 2001). The maintenance and promotion of health is a core feature of wellness tourism (Voigt et al., 2011), which is a form of tourism with a strong health orientation.

The argument that taking part in wellness tourism has health benefits for tourists has been unanimously recognized by many scholars. Tourism experiences are posited to reinvigorate the body and mind, provide opportunities to gain skills, improve self-esteem, and increase awareness of nature (Moscardo, 2009). A healthy wellness tourism experience affects leisure satisfaction, which leads to nonleisure satisfaction, which then helps improve tourist QOL (Neal et al., 1999). Some authors conducted quantitative and qualitative research on the tourism benefits of three types of healthy travelers in Australia and designed six different factors, namely, self-transcendence, physical fitness, escape and relaxation, important people and novelty, self-reinvention, and indulgence, to measure the benefit of healthy tourism (Voigt et al., 2011). From the perspective of self-determination theory, wellness facilities positively affect tourists’ health and enhances tourists’ vitality and mental health (Thal and Hudson, 2019). The above studies suggest that wellness tourism participation may improve various health and wellness measures; however, studies on the effect of wellness tourism experiences on multiple dimensions of health and well-being are lacking. To address this gap, the current study was performed to assess the health benefits of wellness tourism.

## 3. Methodology

### 3.1. Data collection

Resources, such as worldwide peer-reviewed journals from 2002 to 2022 in scientific databases related to the research field (e.g., Science Direct, PubMed, Web of Science), were used in the study. The first step was searching for articles in the database using combinations of the following keywords: “wellness tourism” or “health tourism” and “benefit” or “effect.” The initial keywords were first identified by a panel of experts. The primary literature searches initially yielded 174 results. Next, only review and research articles are retained. Hence, 57 meeting articles, short comments, and covered chapters were excluded. A total of two graduate students generated abstracts that resulted from the search of each word. Lastly, each article was read carefully to confirm its direct relevance to the content of the study, double checked, and filtered; subsequently, eligible articles were classified. After excluding 49 articles with irrelevant content, 68 articles were found eligible for analysis. Additional texts were included in the review based on their historical significance to the understanding of wellness tourism or their ability to provide context to the current discussion. This step allowed the study to focus on the most salient points presented in the articles rather than simply choosing certain parts of the text. After all articles have been evaluated by two graduate students, the coding results were checked every week by two senior experts. The same or similar codes were directly adopted, and the results were determined by experts after discussion for the different codes.

### 3.2. Grounded theory

Grounded theory is a qualitative research method developed by American sociologists Glaser and Strauss. Grounded theory is a simulation and research process of understanding the world from the bottom up. It is a process of constantly raising questions, making comparisons, establishing classifications, establishing connections, and discovering theories (Wertz, 2014). The analysis of data is called coding.

Open coding is a process of data interpretation in which data and phenomena are identified by concepts by disassembling and understanding the text to confirm and develop the concept, making a comparative analysis, and finally extracting the category (Saldaña, 2021). This study is based on a literature review of 68 articles on benefits of wellness tourism from 2002 to 2022. These health benefits of wellness tourism can be extracted through open coding. In this study, the concrete operation of the research method of open coding is realized by the qualitative analysis software Nvivo11.0. After repeated coding and correction, a total of 2,538 reference points were obtained. After repeated comparison and selection, 16 concepts and four main categories were obtained. An example of open encoding is shown in Table 1.

**Table 1:** Opening coding example.

Opening coding example		
Material texts	Conceptualization	Extraction of domain
A study segmenting spagoers in Texas, United States found relaxation to be a notable benefit associated with taking a wellness vacation	Release life stress	Psychological fitness
The notion of transformation, progress, illumination of the mind, and life-changing experiences were present in many of the reviews.	Rejuvenation	
The wellness tourism experience is the antecedent of visitors’ leisure satisfaction, which in turn impacts their nonleisure satisfaction.	Improve life satisfaction	Quality of life
Cope with the normal stresses of life; work productively and fruitfully; make a contribution to one’s community; empower people to incorporate wellness behavior, activities and life habits into their lives.	Realizes one’s self-efficacy	

### 3.3. Framework analysis

This study follows the steps for framework analysis as recommended by Furber (2010). It utilizes a well-defined process based on a large amount of text data, is suitable for the analysis of a large amount of text data in any form, and has systematic rigor for the quality, depth, and richness of the analysis. Nvivo 11.0, a text mining software that supports the analysis of qualitative data, was used to analyze the text.

First, familiarization included reading through the study selected while taking notes on key themes and substantive issues. Here, notes were made of the main ideas that appeared to recur in the data (Ritchie et al., 2013). These recurring ideas from the familiarization process were then collated into groups of similar ideas or themes to be organized into a conceptual framework or index (Ritchie et al., 2013). After a theoretical framework has been developed, the next phase of the framework method is called indexing. This phase involves reading through the data and noting the theme on the draft theoretical framework that it applies to. The final phase of the framework analysis process involves mapping and interpretation to synthesize the data. Here, interpretive codes were explored for homogeneity to assign an overarching theme related to health benefits. A final theoretical framework was then agreed at this stage. At the end of this process, the coding spectrum included four themes and 16 interpretive codes (Table 2).

**Table 2:** Interpretive codes under emergent themes.

Interpretive code	Operational definition of interpretive code
**Physical fitness: The body, organs, and cells of an individual are in good condition**	
Body	Opinions about body function
Sports health	Opinions about a form of wellness activities could be purely physical with a focus on sports and fitness
Prevent disease	Opinions about prevention from illnesses
Healthy nutrition	Opinions about culinary experiences in wellness tourism
**Psychological fitness: The mind, spirit, and emotion are in positive state**	
Emotional wellness	Opinions about positive emotion
Mind control	Opinions about capability to manage one’s feelings and to act accordingly
Rejuvenation	Opinions about rejuvenated state of mind (refreshed/memorable/ enjoyment)
Relaxation	Opinions about the ability to cope with stress
**Quality of life: An overall state of affairs in a particular society that people evaluate positively**	
Self-efficacy	Opinions about realization of one’s own potential
Life satisfaction	Opinions about the emotional status that individuals gain in different areas of life
Well-being	Opinions about different valuations that people make regarding their lives, the events happening to them, their bodies and minds and the circumstances in which they live
Social interaction	Opinions about quality and extend of interaction with others and the community
Engagement	Opinions about a sense of involvement in daily life activities
**Environmental health: Ecological environment and human environment**	
Ecological environment	Opinions about natural environment
Human environment	Opinions about the local atmosphere and folk traditions, which serve as an environmental background

**Figure 1:**
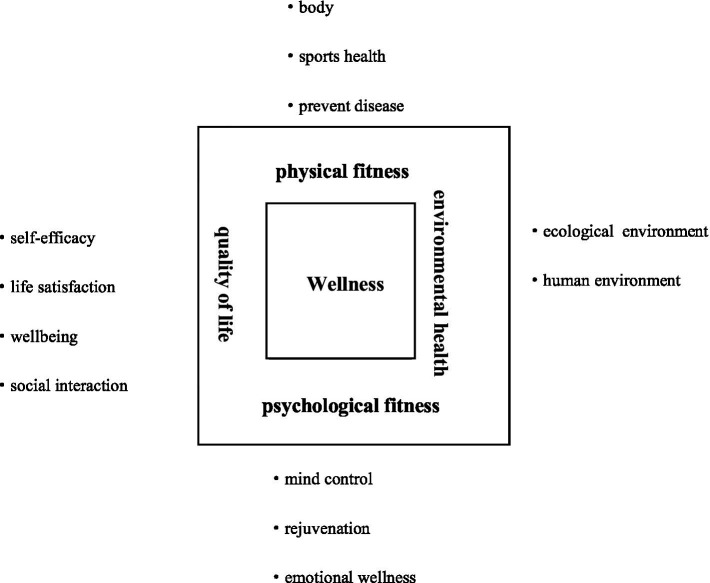
Wellness dimensions of wellness tourism experiences.

Once all charts were complete, a conceptual framework illustrating wellness tourism experiences representing the four dimensions was proposed (Figure 1).

## 4. Results

### 4.1. Physical fitness

Physiological fitness means that the body, organs, and cells of an individual are in good condition (Schilling et al., 2020). Maintaining and promoting good physical health is affected by diet, living environment, and behavioral habits. On this basis, wellness tourism is based on good phenological conditions and promotes the physical and mental health of tourists in the form of tourism to enhance tourist happiness (Filep, 2014). Most tourists who participate in wellness tourism tend to choose a more pleasant climate and a more comfortable natural environment, such as forests, parks, water bodies, and seashores, compared with their permanent residence. People’s behavior in wellness tourism also increases more outdoor environment time and more chances to exercise than usual, allowing people to obtain better health time.

Various scholars have paid attention to studies on the potential impact of the natural environment of wellness tourism on physical health. Following the theory of rehabilitative landscape, some authors argue that natural environment, social interaction, and symbolic landscape affect the healing process of wellness tourists; the level of salivary cortisol, a stress-related physiological marker, is significantly reduced in people who stay longer in green environments (Bell et al., 2018). It also significantly reduces the risk of having type-2 diabetes, cardiovascular disease, early death from hypertension, premature delivery, and stress disorder (Peters, 2000; Li and Kawada, 2011).

A type of tourism that features green environments is forest tourism. Forest tourism is based on forest ecological environment, aiming at promoting public health by using forest ecological resources, landscape resources, food and medicine resources, cultural resources, and the organic integration of medicine and health science to conduct tourist health care, rehabilitation, and health care services (Nara and Horii, 2009). Forest environments are full of negative oxygen ions, plant fungicides, and plant essence. Various health care factors act on the human body, improving immunity, respiratory system function, and cardiovascular health (Liu et al., 2013). Exposure to the forest environment in the winter even induces psychological relaxation (Bielinis et al., 2018).

Forest therapy decreases blood pressure, reduces the pulse rate, and increases parasympathetic nervous activity, which is enhanced in relaxing situations (Ohe et al., 2017). In forested areas, subjects exhibited significantly lower diastolic blood pressure and higher parasympathetic nervous activity but had significantly lower sympathetic nervous activity and heart rate (Tsunetsugu et al., 2013).

Wellness tourism started out as being exclusively linked to the spa sector but is now associated with other tourist products (Ciobanu and Luches, 2018). Spa tourism is regarded as a form of wellness tourism, and various hot spring tourism activities are conducted with hot springs as the carrier (Georgiev and Trifonova Vasileva, 2010). A survey of spa tourists in Victoria, Australia found that 98% and 82% of total respondents believe spas are good for health and sleep, respectively (Clark-Kennedy and Cohen, 2017). Scholars also took the tourists of red ginseng hot springs as the survey object and found that the spa experience can help improve the physical fitness of tourists (Choi et al., 2015). The medical services in wellness tourism play an important role in secondary prevention and preventative healthcare (Huang et al., 2022).

The effect of wellness tourism activities on tourists’ physical health extends to fields, such as medicine and life sciences, and is limited by interprofessional and experimental conditions. Existing research results still lack strong experimental demonstration and mechanism explanation. Only a few studies systematically elaborate the mechanism of healthcare tourism activities on tourists’ physical health.

### 4.2. Psychological fitness

The standard of health is not only on length of life but also on the abundance of the mind. Excessive stress, depression, or anxiety have become prominent problems that plague people in modern society. Psychological fitness is an integral part of human health and involves different mental states, such as attention, stress, and emotion (Folkins et al., 1972). Therefore, there is no health without psychological fitness.

Personal well-being, satisfaction of family and interpersonal relationships, as well as a positive contribution to society are predetermined by psychological fitness. Psychological fitness is an integral part of human health. Unique wellness activities, curriculum training, and interpersonal interaction are conducive to improving the quality of mental health. In forest health tourism, the adjustment and perception of tourists’ psychological state depends on the negative air ions in the forest, plant essence, acoustic environment, quiet environment, natural external irradiation penetrating the radiation dose level, light environment, and tourism microclimate (Hollon et al., 2006). Forest landscapes obtained better scores in subjective ratings and induced significantly less negative and more vigorous moods. Overall, these findings suggest that even a short-term viewing of forests provide relaxing effects (Tsunetsugu et al., 2013). In a study focused on the elderly, participating in wellness tourism was found to improve the sense of dignity and life happiness (Kan et al., 2022).

Scholars conducted psychological tests on tourists who participated in forest tourism through four psychological questionnaires and found that tourists who have short winter interaction with forests experienced substantial emotional, restorative, and vitalizing effects (Bielinis et al., 2018). Furthermore, the mental health benefits of forest health tourism were confirmed by using semantic discrimination and subjective symptom index methods. In forest tourism activities, the design of leisure activities is particularly important (Ohe et al., 2017). Psychological guidance forest leisure tourism products can effectively improve the happiness index and psychological state of tourists (Park et al., 2011).

Rural green tourism also has a wellness purpose and is one of the subtypes of wellness tourism. Notably, mental improvement and rehabilitation can and should be conducted within rural green tourism, leading to a positive impact on mental and overall human health. On the basis of the perspective of restorative environment, the restorative benefits of rural wellness tourism destinations and the restorative experience of tourists are studied, and the psychological recovery effect of wellness tourism on individual tourists is determined (Soloviov et al., 2019). The kinds of wellness activities in contact with nature positively impact tourist psychological fitness.

According to environmental psychology, numerous experimental studies have demonstrated that wellness tourism activities in the natural environment improve the mental health of tourists in the short term, but it is uncertain whether such mental health plays a positive role in the long term (Croes et al., 2018). Therefore, the impact of wellness tourism activities on tourists’ mental health is still a direction that needs to be further explored in academic discussions.

### 4.3. Quality of life

Different disciplines have different definitions of QOL. Development economics and sociology suggest that it means the significance of mental life level to people and social development. Psychologists believe that subjective well-being is happiness in the sense of QOL, which forms the measurement of happiness in happiness research (Carlson et al., 2003; Cruice et al., 2003). The World Health Organization defines QOL as reflecting personal goals, expectations, standards, awareness, and views on life.

Scholars have different definition of QOL, but a consensus exists—it has subjective feelings and objective evaluation. Objective QOL emphasizes people’s material living conditions and evaluates people’s QOL from the objective aspects that affect people’s material and spiritual life. The guiding conceptual principle of QOL is composed of “those same factors and relationships for people with special needs that are important to everyone” and applies the core principle that “QOL application should enhance a person’s well-being” (Helm, 2003).

Tourists following healthy lifestyle principles are more interested in visiting wellness facilities (Kim et al., 2015) and are likelier to choose health promotion-related and preservation-related products and services (Hallab, 2006). Education, esthetics, and escapism in wellness tourism are closely related to the tourist experience, which increases tourist satisfaction and improves overall QOL (Luo et al., 2018). Scholars in the field of wellness tourism also pointed out that the experience of such tourism activities is positively correlated with QOL (Koncul, 2012). For example, health facilities in forest tourism make tourists better feel the ecological environment, enhance interaction, and improve interpersonal relationships (Qin and Cheng, 2021).

The product characteristics of health tourism also affect the tourism experience and life satisfaction of the elderly, as evidenced by empirical research (Kan et al., 2022). The more satisfied older travelers are with their travel experience, the better their overall QOL (Kim et al., 2015). Findings of well-being research on tourism settings are supported by establishing the importance of social, intellectual, and spiritual dimensions of tourists’ lifestyle (Težak Damijanić, 2019). The retreat experience helps participants gain control over their life and make positive adjustments to their lifestyle, which leads to health improvements that continued after they returned to their regular routines (Cohen et al., 2017). Evidently, the physical and mental well-being produced by wellness tourism activities often results in individuals feeling calmer, more energetic, and more enthusiastic and can improve problem solving at work, promote family harmony, and participation in social life.

The improvement of QOL is an external manifestation of participating in wellness tourism. Existing research on the health benefits of wellness tourism is mostly from the perspective of tourists’ perception, and the impact of wellness tourism experience on life satisfaction is subjectively judged, thereby lacking quantitative empirical study to determine the impact of wellness tourism on QOL.

### 4.4. Environmental health

Environmental health refers to the ecological environment composed of air, water, soil, noise, and the human environment composed of language and traditional customs, which can meet the needs of the people and society for healthy development. Research on the environmental health benefits of wellness tourism is conducted from two aspects: ecological environment and human environment.

On the one hand, scholars study the health benefits of the ecological environment. Destinations that develop tourism fail to maintain ecological and environmental health, delivering negative knock-on effects (Loehr et al., 2022). From the ecology perspective, wellness tourism refers to the realization of a higher-level dynamic balance between the individual tourist’s microecosystem and other natural and social ecosystems in the process of complex material, energy, and information exchanges (Luo et al., 2022). The construction of wellness tourism destinations requires a comprehensive assessment of the regional tourism environment. Developing wellness tourism in an area can create quality products and promote environmental protection (Sheldon and Park, 2008).

On the other hand, scholars study the health benefits of the human environment. Culture plays an important role in molding relationships between place and health. During the construction of health tourism destinations, local historical and cultural resources are incorporated, showing character and personality, enhancing the sense of cultural pride and identity of residents and tourists. The symbolic landscape, which is deeply shaped by the longevity culture, plays a dominant role in the development of wellness tourism in Bama, one of the world’s top five “longevity” villages (Huang and Xu, 2018). The distinctive seaside, forest, grassland, lakeside, and other natural environments are accompanied by different local customs and dining characteristics. The unique humanistic attribute of different regions is a necessary part for the development of regional wellness tourism industry. It plays an important role in local economic reconstruction, image shaping, historical and cultural identity, as well as the identification of tourism products and services (Taff et al., 2019).

## 5. Conclusion and implications

On the basis of the mainstream database, this study reviews the literature on the health benefits of wellness tourism, which further enhances the systematization and integrity of the theory. From the existing research results, this study draws the following conclusions:

First, four main dimensions namely, physical fitness, psychological fitness, QOL, and environmental health, were extracted.

Second, in the dimension of the body, previous studies on the health benefits of wellness tourism have conceptually classified wellness tourism as a new tourism model (Koncul, 2012) and have qualitatively analyzed the overall health dimension brought by the wellness tourism experience based on tourism reviews (Dillette et al., 2021). Multidisciplinary and multiperspective research on the physiological health benefits of wellness tourism, as well as more reliable experimental data and more scientific qualitative research to explore the impact of wellness tourism activities on physical health, are lacking. For high-level health tourists, groups with diet and health orientation, fitness orientation, and low-level health, the health maintenance and promotion effects in the wellness tourism environment vary (Težak Damijanić, 2019).

Third, unique wellness tourism is conducive to improving the quality of psychological fitness. For people with rehabilitation orientation, the long-term impact of the rehabilitation experience gained from staying in a healthy hotel and participating in wellness activities is deeper (Kim and Yang, 2021). Given that the human body is a complex network open system, the differences between individual tourists make the health benefits of tourism activities vary, making it difficult to measure by a unified standard. Therefore, in the future, different groups must be chosen as research objects to explore the establishment of tourism activities as nondrug therapy for human health intervention. Lastly, participating in wellness tourism not only improves QOL but also brings environmental health, including ecological environment and human environment.

With the increasing awareness of health and the need to relieve work stress, people increasingly consider participating in wellness tourism to maintain and improve their health. Health is a multidimensional concept based on balance and spirit, combining physical and mental health and social and environmental factors. The current research on health tourism focuses on promoting and improving the effects of tourism on human health. The findings have important theoretical and practical implications for academics, managers, and enterprises in the tourism industry, which indicate that the positive effects of wellness tourism experiences on perceived health and wellness have been demonstrated by multiple studies.

### 5.1. Theoretical implications

In combination with the general health background, this study discusses the health benefits brought by participating in wellness tourism from an overall perspective, thereby enriching the research direction of health tourism and emphasizing the significance of health benefits generated by wellness tourism; thus, this study can provide reference for subsequent research on wellness tourism.

1. The influencing factors of wellness tourism must be studied further. Recent studies have focused on the impact of landscape and natural environment. The exploration of impact factors can guide the planning and design of tourism destinations based on health orientation.
2. The effects of wellness tourism on health benefits still need to be explored. The most recognized ways of acting are to encourage physical activity, relieve stress, promote social interaction, and provide medical services. The specific mechanism of action must be studied in the future. For example, the mental aspect of wellness tourism should be highlighted and exposed as a mechanism by which participants can experience holistic wellness. The outcome of wellness tourism is intangible and subjective; thus, visitors’ comments, as well as long-term dynamic observation, must be focused on to show the benefits of wellness tourism.
3. Research methods must be updated. The existing methods are still mainly interviews and questionnaires of traditional social sciences, and these methods rarely integrate with natural science methods, such as medicine and environmental science. Therefore, how to realize the complementarity of multidisciplinary methods and create a measurement method with scale and precision is worth deepening. Follow-up research can also be combined with emerging technologies, such as the use of “big data crawling,” to obtain residents’ emotional health values and the use of virtual reality technology to achieve natural interactive operation.

### 5.2. Managerial implications

All tourism services, from tourism planning, product marketing, tourism reception to tourism management, should be closely focused on creating unique health benefits.

1. A focus on creating and sustaining an environment conducive to wellness is paramount to wellness tourism providers. When it comes to the supply system and main components, wellness tourism involves many factors, such as food, sports, culture, and physical and mental nursing (Dini and Pencarelli, 2022). Therefore, in the design of wellness tourism, the construction of multiple functions, such as sports, leisure, entertainment, business, and social interaction, should be strengthened to improve the efficiency of health transformation and improve QOL. Wellness tourism destinations and scenic spots, from the technical and practical levels, should adopt a health management model or a professional health maintenance plan to achieve the perfect combination of “health protection” and “health management” and thus enhance the tourist experience (Kim et al., 2017).
2. When planning wellness tourism destinations, the plant landscape must be enriched to improve the natural elements and the health benefits of space to relieve mental stress. Moreover, the sense of space experience must be enhanced, a comfortable environment of healthy space must be created, and social interaction must be promoted. Each tourist destination has its own characteristics, including humanities, nature, society, and environment. The development of tourist destinations requires unique attraction. Therefore, tourism managers should develop local characteristic resources, provide professional health services, to make a difference with other tourist destinations.
3. For wellness tourism destination marketing, to create a holistic sense of wellness for travelers, four main strategies based on the revealed dimensions should be prioritized for the sustained development of the wellness tourism industry. From a marketing perspective, wellness tourism’s services and products that match customers’ needs and preferences must be understood. Some research posit that relaxation and recovery are key motivations for participating in wellness tourism (Voigt et al., 2011), and personal attention to health and life stress are important antecedents (Thal and Hudson, 2019). Therefore, the unique environment and health activities of wellness tourism should be highlighted in the publicity materials, with the health benefits that wellness tourism can achieve explicitly conveyed, so that tourists can understand the effect of healing their psychological and physical diseases. This improves their QOL through participation in wellness tourism, stimulating the desire for taking part in wellness tourism.

### 5.3. Policy implications

The government should highlight the transformation of scientific research and practice. As for the research on health benefits of wellness tourism, scientific research disciplines, such as environmental science and medicine, are in a state of disconnection with practice disciplines, such as planning and design, landscape design and forestry. Communication platforms and cooperation mechanisms should be built between different disciplines to transform rich scientific research achievements into health tourism practice and application to maximize health benefits.

### 5.4. Future research and limitation

This study enriched the understanding of wellness tourism experiences that help in the planning and management of wellness tourism. However, it has some limitations. Given the nature of the data collected for this study, a few new forms of wellness tourism have not been explored. Additionally, as this study is an open exploratory study using grounded theory, no deductive analysis has been conducted in conjunction with classical theory. Future research can be based on the framework of this study, and dynamic extrapolation in conjunction with self-regulation theory and attention recovery theory can be conducted to gain a deeper understanding of the health benefits of wellness tourism.

## Author contributions

CL contributed to the conception of the study and contributed significantly to analysis and manuscript preparation. CL and SX collected and organized the data, performed the data analyses, and wrote the manuscript. YZ, RL, and MZ helped perform the analysis with constructive discussions. MZ was responsible for the overall project. All authors contributed to the article and approved the submitted version.

## Conflict of interest

The authors declare that the research was conducted in the absence of any commercial or financial relationships that could be construed as a potential conflict of interest.

## Publisher’s note

All claims expressed in this article are solely those of the authors and do not necessarily represent those of their affiliated organizations, or those of the publisher, the editors and the reviewers. Any product that may be evaluated in this article, or claim that may be made by its manufacturer, is not guaranteed or endorsed by the publisher.
